# Correction to ‘Augmenting disease maps: a Bayesian meta-analysis approach’

**DOI:** 10.1098/rsos.210085

**Published:** 2021-02-10

**Authors:** Farzana Jahan, Earl W. Duncan, Susanna M. Cramb, Peter D. Baade, Kerrie L. Mengersen

*R. Soc. Open Sci.* **7**, 192151 (Published 5 August 2020) (doi:10.1098/rsos.192151)

This correction refers to an error in the numeric values reported in table 4, which is a subset of table 13. The correct values are displayed below.

This error has no implications on the results and conclusions drawn in the publication. The paper has now been updated.

**Table 4.** Pairwise comparison of region specific mean for lung cancer.

	probability	
pairs	male	female
major cities > regional	0.000	0.001
major cities > remote	0.000	0.006
regional > remote	0.0001	0.061

