# Supplementary material for: Augmenting disease maps: a Bayesian meta-analysis approach
Source: R Soc Open Sci. 2020 Aug 5;7(8):192151. doi: 10.1098/rsos.192151 (PMC7481717; doi:10.1098/rsos.192151)
Supplement: Supplementary Material [file rsos192151supp1.pdf]

ROYAL SOCIETY
OPEN SCIENCE

rsos.royalsocietypublishing.org

Research

CrossMark
click for updates

Article submitted to journal

Author for correspondence:
Farzana Jahan,
e-mail: f.jahan@hdr.qut.edu.au

Supplementary Material for Augmenting Disease Maps: a Bayesian meta-analysis approach

Farzana Jahan [1], Earl W. Duncan [1], Susanna M. Cramb [2], Peter D. Baade [2] and Kerrie L. Mengersen[1]

[1]School of Mathematical Science, ARC Centre of Excellence for Mathematical and Statistical Frontiers, Queensland University of Technology, Australia
[2]Cancer Council Queensland, Brisbane, Queensland, Australia

© 2014 The Authors. Published by the Royal Society under the terms of the Creative Commons Attribution License http://creativecommons.org/licenses/by/4.0/, which permits unrestricted use, provided the original author and source are credited.

THE ROYAL SOCIETY
PUBLISHING

1. Sensitivity Analysis

To make appropriate choices of priors for the proposed Bayesian hierarchical meta-analysis model (Section 3.1, equation 1-8 in the paper), a rigorous sensitivity analysis was carried out. The sensitivity analysis was performed using modelled estimates from Australian Cancer Atlas (ACA) for all cancers combined (for all persons), lung cancer for female, and some rare cancer types, such as, oesophageal cancer for females, liver cancer for females and thyroid cancer for males. Our goal was to choose a sensible prior combination which would perform reasonably well for all the different cancers (common or rare). The assessment criteria for the prior combinations are briefly discussed in section 2.

The proposed Bayesian hierarchical meta-analysis model assumes a normal distribution for $Y_{ij} = log(SIR)$ for $i = 1, 2, ..., 2148$ small areas (SA2's) with mean μ_{ij} and a variance σ^2_{ij}. The prior for σ^2_{ij} is chosen to be a χ^2_ν with a small degrees of freedom ($\nu = 2$) utilising the associated standard deviations with the SIR estimates reported in the ACA (Section 3.1.1 in the article). The μ_{ij} is modelled as a Normal distribution with a mean θ_j, $j = 1, 2, 3$ remoteness regions and region-specific variances σ^2_j. The region specific means θ_j is further modelled as a Normal distribution with mean μ_0, which is an overall mean across Australia for the cancer under consideration and an overall variance parameter σ^2_0. The sensitivity analysis was performed to choose appropriate priors and hyperparamters for the overall mean (μ_0), the variance parameters (σ^2_j and σ^2_0) or the precision parameters ($\tau_j = 1/\sigma^2_j$ and $\tau_0 = 1/\sigma^2_0$).

The priors considered for the overall mean, μ_0, as well as for the variance parameters, σ^2_j for $j = 1, 2, 3$ remoteness regions and σ^2_0 or the precision parameters, $\tau_j = 1/\sigma^2_j$, and $\tau_0 = 1/\sigma^2_0$, where σ^2_j and σ^2_0 are within region variances and overall variance respectively.

(a) Prior for overall mean

The convention of choosing a prior for the overall mean in a Bayesian hierarchical model suggests use of a normal prior with zero mean and a very large variance. However the modern literature suggests that while choosing a prior, we also need to think of the data to which the model is going to be applied **?**. Since the estimated $log(SIR)$ values from ACA vary between -4 to 2 and the extreme values are very rare, the use of a very large prior variance for the overall mean $log(SIR)$ for these data does not seem to be reasonable. Hence a sensitivity analysis is performed to obtain a suitable value of hyperparameter for prior of the overall mean in the proposed model.

The prior for the overall mean was specified as:

$$\mu_0 \sim N(0, var) \tag{1.1}$$

and different values for the variance hyperparameter var are compared. The choices of var used in the sensitivity analysis were $2, 5, 10$ and 100.

(b) Priors for the variance and/or precision parameters

The choice of priors for variance parameters in a Bayesian hierarchical model has been of great interest in the literature **??**. The most common practice is to specify inverse Gamma priors for the variance parameters which is a conjugate distribution of the normal distribution **?**. However some alternatives have been suggested which might give better model performance **?????**. These include a half-normal prior for the standard deviation **?**, a hierarchical prior for the variance **?** and a Bayesian Lasso prior **?**. All these different suggestions were compared for the proposed meta-analysis model. We also explored specifying half-normal priors for the precision parameters instead of the standard deviation.

The different priors considered in this sensitivity analysis were:

Inverse Gamma Priors

$$\sigma_0^2 \sim IG(\alpha_1, \alpha_2) \tag{1.2}$$

$$\sigma_j^2 \sim IG(\alpha_1, \alpha_2) \tag{1.3}$$

The different combinations of shape and scale parameters, denoted by α_1 and α_2 respectively, under comparison were: $(1, 1)$, $(0.1, 0.1)$, $(0.1, 0.01)$, $(0.01, 0.1)$, $(0.01, 0.01)$, $(0.01, 0.001)$, $(0.001, 0.01)$ and $(0.001, 0.001)$.

Half-normal prior for standard deviation
Half-Normal priors (Normal distribution truncated below at zero, denoted by N^+) for the standard deviation parameters of the proposed models were specified as:

$$\sigma_0 \sim N^+(0, prec) \tag{1.4}$$

$$\sigma_j \sim N^+(0, prec) \tag{1.5}$$

The choices of precision ($1/variance$) hyperparameter $prec$ compared were: $0.001, 0.01, 0.1$ and 1.
Hierarchical prior and Bayesian Lasso for variance parameters
The priors specified below were also applied for the region specific variance parameter σ_j^2.

$$\sigma_0^2|\epsilon_0^2 \sim G(\frac{1}{2}, \frac{1}{2\epsilon_0^2}) \Leftrightarrow \sigma_0|\epsilon_0^2 \sim N(0, \epsilon_0^2) \tag{1.6}$$

$$\epsilon_0^2|a^\epsilon, \kappa^2 \sim G(a^\epsilon, a^\epsilon \kappa^2/2) \tag{1.7}$$

$$\kappa^2 \sim G(d_1, d_2) \tag{1.8}$$

We considered the following priors with specific values for the hyperparameters as $d_1 = d_2 = 1$:

Hierarchical prior with

$$a^\epsilon \sim Exp(10) \tag{1.9}$$

and Bayesian Lasso prior with

$$a^\epsilon = 1 \tag{1.10}$$

Half-Normal Priors for Precision parameters
Half-Normal priors for the precision parameters of the proposed models were specified as:

$$\tau_0 \sim N^+(0, prec) \tag{1.11}$$

$$\tau_j \sim N^+(0, prec) \tag{1.12}$$

The choices of precision hyperparameter $prec$ were: $0.001, 0.01, 0.1$ and 1.

(c) Combinations of priors

The different combinations of priors derived using the above-mentioned prior distributions are shown in the Tables 1-5.

Table 1. Prior combinations using Inverse Gamma for variance parameters

	Priors		
Combination	$\mu_0 \sim N(0, var)$	$\sigma_0^2 \sim IG(\alpha_1, \alpha_2)$	$\sigma_j^2 \sim IG(\alpha_1, \alpha_2)$
1	$var = 2$	$\alpha_1 = 1, \alpha_2 = 1$	$\alpha_1 = 1, \alpha_2 = 1$
2		$\alpha_1 = 0.1, \alpha_2 = 0.1$	$\alpha_1 = 0.1, \alpha_2 = 0.1$
3		$\alpha_1 = 0.1, \alpha_2 = 0.01$	$\alpha_1 = 0.1, \alpha_2 = 0.01$
4		$\alpha_1 = 0.01, \alpha_2 = 0.1$	$alpha_1 = 0.01, \alpha_2 = 0.1$
5		$\alpha_1 = 0.01, \alpha_2 = 0.01$	$\alpha_1 = 0.01, \alpha_2 = 0.01$
6		$\alpha_1 = 0.01, \alpha_2 = 0.001$	$\alpha_1 = 0.01, \alpha_2 = 0.001$
7		$\alpha_1 = 0.001, \alpha_2 = 0.01$	$\alpha_1 = 0.001, \alpha_2 = 0.01$
8		$\alpha_1 = 0.001, \alpha_2 = 0.001$	$\alpha_1 = 0.001, \alpha_2 = 0.001$
9	$var = 5$	$\alpha_1 = 1, \alpha_2 = 1$	$\alpha_1 = 1, \alpha_2 = 1$
10		$\alpha_1 = 0.1, \alpha_2 = 0.1$	$\alpha_1 = 0.1, \alpha_2 = 0.1$
11		$\alpha_1 = 0.1, \alpha_2 = 0.01$	$\alpha_1 = 0.1, \alpha_2 = 0.01$
12		$\alpha_1 = 0.01, \alpha_2 = 0.1$	$alpha_1 = 0.01, \alpha_2 = 0.1$
13		$\alpha_1 = 0.01, \alpha_2 = 0.01$	$\alpha_1 = 0.01, \alpha_2 = 0.01$
14		$\alpha_1 = 0.01, \alpha_2 = 0.001$	$\alpha_1 = 0.01, \alpha_2 = 0.001$
15		$\alpha_1 = 0.001, \alpha_2 = 0.01$	$\alpha_1 = 0.001, \alpha_2 = 0.01$
16		$\alpha_1 = 0.001, \alpha_2 = 0.001$	$\alpha_1 = 0.001, \alpha_2 = 0.001$
17	$var = 10$	$\alpha_1 = 1, \alpha_2 = 1$	$\alpha_1 = 1, \alpha_2 = 1$
18		$\alpha_1 = 0.1, \alpha_2 = 0.1$	$\alpha_1 = 0.1, \alpha_2 = 0.1$
19		$\alpha_1 = 0.1, \alpha_2 = 0.01$	$\alpha_1 = 0.1, \alpha_2 = 0.01$
20		$\alpha_1 = 0.01, \alpha_2 = 0.1$	$alpha_1 = 0.01, \alpha_2 = 0.1$
21		$\alpha_1 = 0.01, \alpha_2 = 0.01$	$\alpha_1 = 0.01, \alpha_2 = 0.01$
22		$\alpha_1 = 0.01, \alpha_2 = 0.001$	$\alpha_1 = 0.01, \alpha_2 = 0.001$
23		$\alpha_1 = 0.001, \alpha_2 = 0.01$	$\alpha_1 = 0.001, \alpha_2 = 0.01$
24		$\alpha_1 = 0.001, \alpha_2 = 0.001$	$\alpha_1 = 0.001, \alpha_2 = 0.001$
25	$var = 100$	$\alpha_1 = 1, \alpha_2 = 1$	$\alpha_1 = 1, \alpha_2 = 1$
26		$\alpha_1 = 0.1, \alpha_2 = 0.1$	$\alpha_1 = 0.1, \alpha_2 = 0.1$
27		$\alpha_1 = 0.1, \alpha_2 = 0.01$	$\alpha_1 = 0.1, \alpha_2 = 0.01$
28		$\alpha_1 = 0.01, \alpha_2 = 0.1$	$alpha_1 = 0.01, \alpha_2 = 0.1$
29		$\alpha_1 = 0.01, \alpha_2 = 0.01$	$\alpha_1 = 0.01, \alpha_2 = 0.01$
30		$\alpha_1 = 0.01, \alpha_2 = 0.001$	$\alpha_1 = 0.01, \alpha_2 = 0.001$
31		$\alpha_1 = 0.001, \alpha_2 = 0.01$	$\alpha_1 = 0.001, \alpha_2 = 0.01$
32		$\alpha_1 = 0.001, \alpha_2 = 0.001$	$\alpha_1 = 0.001, \alpha_2 = 0.001$

Table 2. Prior combinations using half-normal for Standard deviations

	Priors		
Combination	$\mu_0 \sim N(0, var)$	$\sigma_0 \sim N^+(0, prec)$	$\sigma_j^2 \sim N^+(0, prec)$
33	$var = 2$	$prec = 0.001$	$prec = 0.001$
34		$prec = 0.01$	$prec = 0.01$
35		$prec = 0.1$	$prec = 0.1$
36		$prec = 0.5$	$prec = 0.5$
37	$var = 5$	$prec = 0.001$	$prec = 0.001$
38		$prec = 0.01$	$prec = 0.01$
39		$prec = 0.1$	$prec = 0.1$
40		$prec = 0.5$	$prec = 0.5$
41	$var = 10$	$prec = 0.001$	$prec = 0.001$
42		$prec = 0.01$	$prec = 0.01$
43		$prec = 0.1$	$prec = 0.1$
44		$prec = 0.5$	$prec = 0.5$
45	$var = 100$	$prec = 0.001$	$prec = 0.001$
46		$prec = 0.01$	$prec = 0.01$
47		$prec = 0.1$	$prec = 0.1$
48		$prec = 0.5$	$prec = 0.5$

Table 3. Prior combinations using Hierarchical prior for variance parameters

	Priors		
Combination	$\mu_0 \sim N(0, var)$	$\sigma_0^2 \sim$Hierarchical Prior	$\sigma_j^2 \sim$ Hierarchical Prior
49	$var = 2$	Equation 6-10, with $d_1 = d_2 = 1$	Equation 6-10, with $d_1 = d_2 = 1$
50	$var = 5$		
51	$var = 10$		
52	$var = 100$		

Table 4. Prior combinations using Bayesian Lasso for variance parameters

	Priors		
Combination	$\mu_0 \sim N(0, var)$	$\sigma_0^2 \sim$ Bayesian Lasso	$\sigma_j^2 \sim$ Bayesian Lasso
53	$var = 2$	Equation 6-9,11, with $d_1 = d_2 = 1$	Equation 6-9, 11, with $d_1 = d_2 = 1$
54	$var = 5$		
55	$var = 10$		
56	$var = 100$		

Table 5. Prior combinations using half-normal for Precision Parameters

	Priors		
Combination	$\mu_0 \sim N(0, var)$	$\tau_0 \sim N^+(0, prec)$	$\tau_j \sim N^+(0, prec)$
57	$var = 2$	$prec = 0.001$	$prec = 0.001$
58		$prec = 0.01$	$prec = 0.01$
59		$prec = 0.1$	$prec = 0.01$
60		$prec = 0.01$	$prec = 0.1$
61		$prec = 0.1$	$prec = 0.1$
62	$var = 5$	$prec = 0.001$	$prec = 0.001$
63		$prec = 0.01$	$prec = 0.01$
64		$prec = 0.1$	$prec = 0.01$
65		$prec = 0.01$	$prec = 0.1$
66		$prec = 0.1$	$prec = 0.1$
67	$var = 10$	$prec = 0.001$	$prec = 0.001$
68		$prec = 0.01$	$prec = 0.01$
69		$prec = 0.1$	$prec = 0.01$
70		$prec = 0.01$	$prec = 0.1$
71		$prec = 0.1$	$prec = 0.1$
72	$var = 100$	$prec = 0.001$	$prec = 0.001$
73		$prec = 0.01$	$prec = 0.01$
74		$prec = 0.1$	$prec = 0.01$
75		$prec = 0.01$	$prec = 0.1$
76		$prec = 0.1$	$prec = 0.1$

2. Some Selected Outputs from the Sensitivity Analysis

Each of the 76 prior combinations showed in the previous section was used to fit the proposed Bayesian hierarchical meta-analysis model using some common cancers (all cancers combined for persons and lung cancer for females) and some more rare cancers (Liver cancer, Oesophageal cancer for females and Thyroid cancer for males). For each of the model combinations, 3 parallel MCMC chains were run with 100000 iterations each with a burn in period of 10000 iterations. The most suitable model was chosen on the basis of visual diagnostics (density and trace plots for the parameters of interest), posterior predictive checks, mean squared error (MSE), average bias and deviance information criteria (DIC). In the event that no unique best model was identified according to all the criteria, emphasis was put on convergence characteristics (visual diagnostics and Gelman Rubin Statistic **?**) and acceptable performance on the other criteria.

In this section, we report some results using one combination from each of the 5 tables for illustration purposes. All 76 prior combinations were evaluated to choose the suitable model.

Table 6. Model Diagnostics for selected Prior combinations for selected cancers

Prior Combination	Cancer	DIC	Avg (Bias)	MSE
8	All cancer (persons)	358.736	0.0052	0.059
41	Lung Cancer(female)	346.01	0.0062	0.082
49	All cancer (persons)	-4014.917	-0.0015	0.0048
	Liver cancer (female)	-1003.09	0.0038	0.0317
54	Oesophageal cancer (female)	-992.91	0.0056	0.0675
75	Thyroid cancer (male)	239.5004	-0.0034	0.026

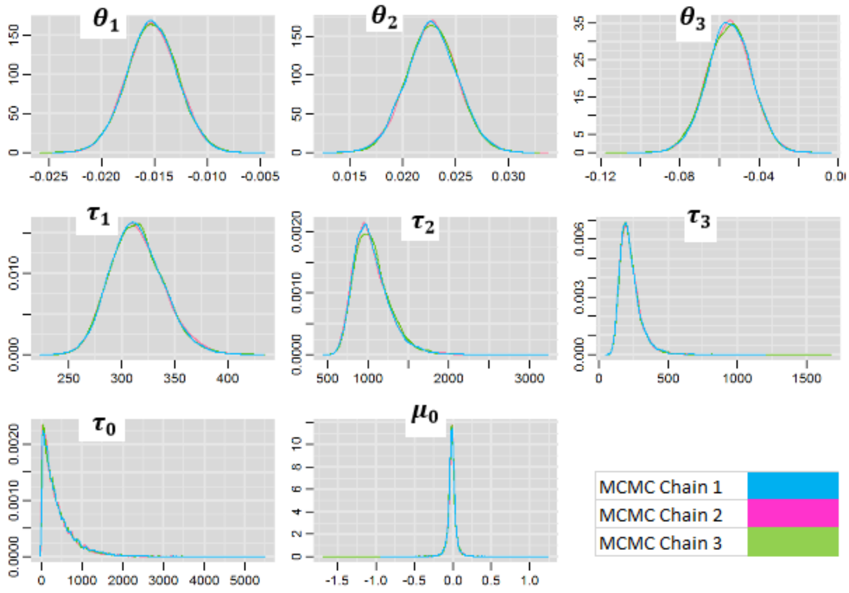

Figure 1. Posterior Density plots using prior combination 8 (Inverse Gamma priors for variances) for all cancers combined (all persons)

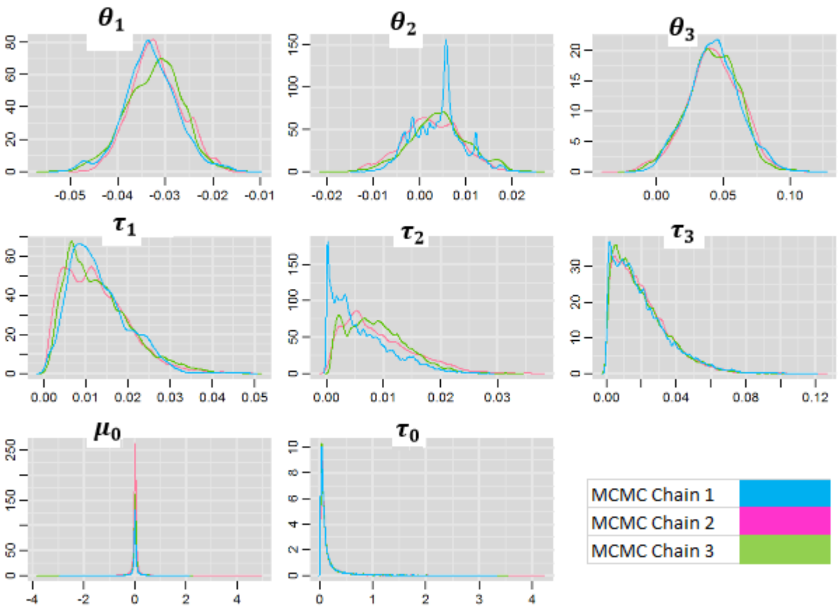

Figure 2. Posterior Density plots using prior combination 41 (half-normal priors for standard deviations) for lung cancer(females)

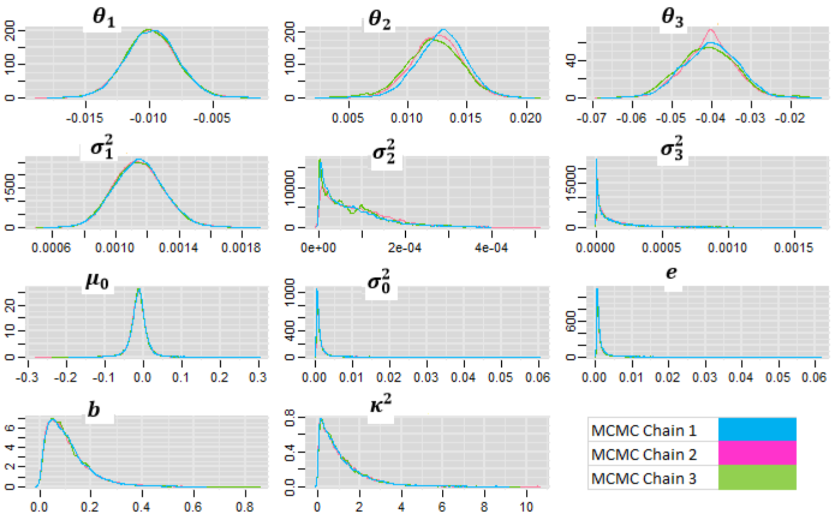

Figure 3. Posterior Density plots using prior combination 49 (Hierarchical priors for variances) for all cancers combined (all persons)

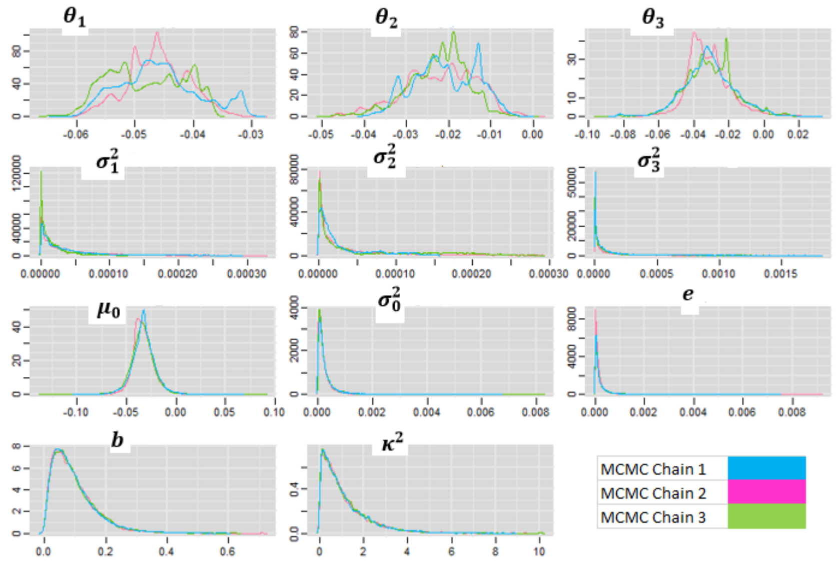

Figure 4. Posterior Density plots using prior combination 49 (Hierarchical priors for variances) for liver cancer (females)

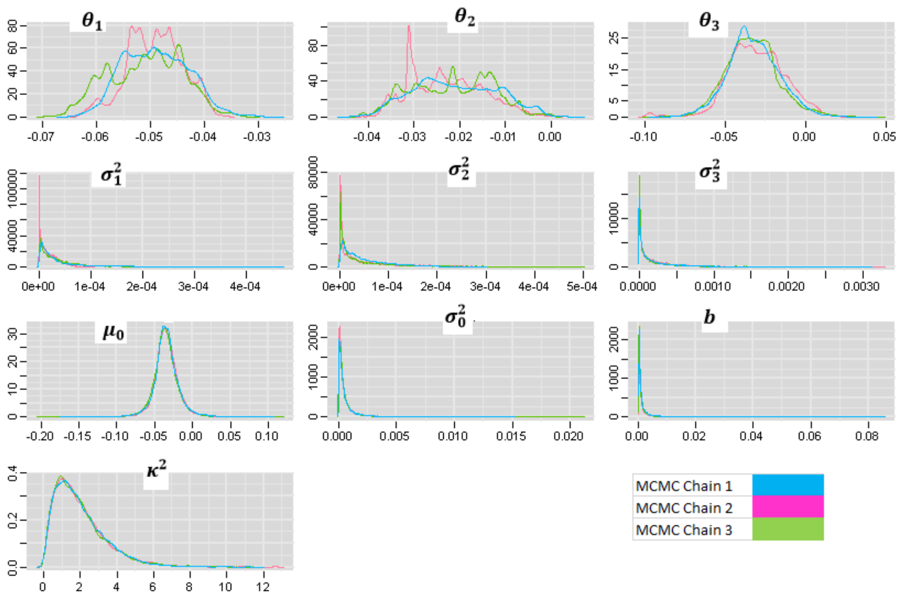

Figure 5. Posterior Density plots using prior combination 54 (Bayesian Lasso prior for variances) for oesophageal cancer(females)

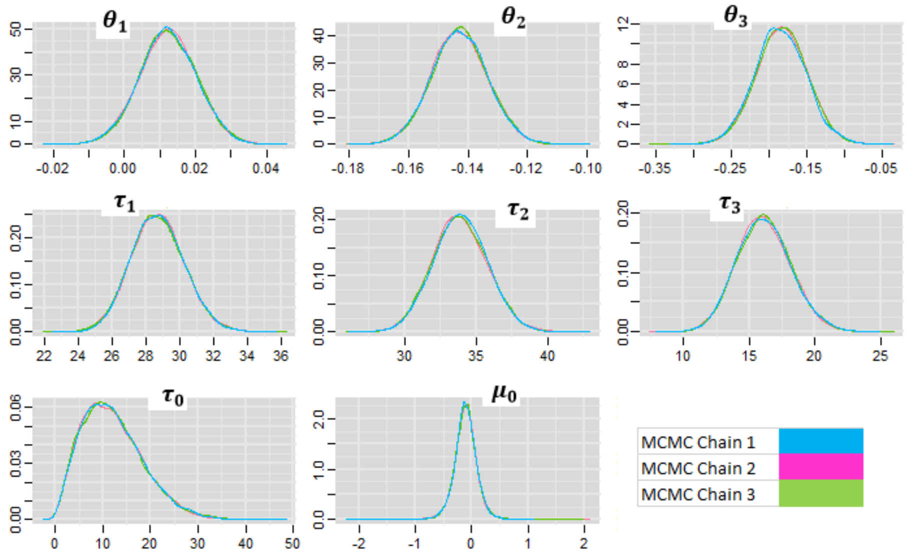

Figure 6. Posterior Density plots using prior combination 75 (half-normal Prior for precisions) for thyroid cancer (males)

3. Results and Discussion

The above results and those obtained from evaluation of all 76 prior combinations are discussed in this section, along with the final choice of priors for the proposed model.

Figure 1 shows the posterior density plots of region specific means, precision parameters, overall mean and precision from the fitted MCMC results using prior combination 8 (Table 1), which assigned Inverse Gamma priors for region-specific variances and the overall variance parameter. The results are shown for all cancers combined for all persons. Similar results were obtained using the other combinations specified in Table 1 for other common and rare cancer types. Although the prior choice does not show poor performance, there are valid criticisms against using the inverse Gamma distribution with small scale and shape parameters **?**. Moreover, in our sensitivity analysis, other combinations showed improved performance, as discussed below.

Figure 2 shows the posterior density plots of parameters associated from the fitted MCMC results with prior combination 41 (Table 2), which assigned half-normal priors for the region-specific standard deviations and the overall standard deviation parameter. Visually, the density plots show poor convergence of the model. Similar results were obtained using the other combinations of hyperparameters for this prior distribution specified in Table 2.

Figures 3 and 4 show the posterior density plots of parameters associated with prior combination 49 (Table 3) for all cancer combined and for a rarer cancer type respectively, which assigned hierarchical priors for the region-specific variances and the overall variance parameter. Based on Figure 3, the model appears to perform well for all cancers combined, but from Figure 4, it is seen that the model is not a good choice for rarer cancers. Similar results were obtained using the other rarer cancers.

Figure 5 shows the posterior density plots of parameters associated with combination 54 (Table 4), which assigned a Bayesian lasso prior for the region-specific variances and the overall variance parameter. The density plots indicate unsatisfactory performance for these priors. Similar observations were found for all other rare and common cancer types for the other hyperparameter combinations specified in Table 4.

Figure 6 shows the posterior density plots of parameters associated with the prior combination 75 (Table 5), which assigned half-normal priors for the region-specific precision parameters and the overall precision parameter. The result is shown for thyroid cancer for males, which is a rarer cancer type. The prior combinations specified in Table 5 behaved reasonably well for all cancer types considered in this sensitivity analysis.

The other diagnostic measures were consistent with the results of the visual output shown here. In particular, specifying half-normal priors for precision parameters was a very good choice in terms of satisfactory model performance.

Until now, we have only emphasised the choice of priors for variance or precision parameters. However these combinations have different hyperparameters for the normal prior specified for the overall mean as well. The overall mean, being the last layer of the Bayesian hierarchical meta-analysis model, is not very sensitive to the choice of hyperparamter of its prior. Hence, we chose the hyperparameter, which is the variance of the Normal prior, to be 100, to allow for good coverage of anticipated values and based on inspection of the data. Hence, the prior combination we specified for the proposed Bayesian hierarchical meta-analysis model in the article is prior combination 75 (section 3.1, equations 4, 6 & 7 in the paper), which was chosen by examining the outputs of all 76 prior combinations for different cancer types.

References

[1] Miguel AG Belmonte, Gary Koop, and Dimitris Korobilis. "Hierarchical shrinkage in time-varying parameter models". In: *J Forecast.* 33.1 (2014), pp. 80–94. URL: https://doi.org/10.1002/for.2276.

[2] Angela Bitto and Sylvia Frühwirth-Schnatter. "Achieving shrinkage in a time-varying parameter model framework". In: *J Econom.* (2018). URL: https://doi.org/10.1016/j.jeconom.2018.11.006.

[3] Sylvia Frühwirth-Schnatter. "Efficient Bayesian parameter estimation for state space models based on reparameterizations". In: *State Space and Unobserved Component Models: Theory and Applic.* (2004), pp. 123–151.

[4] Sylvia Frühwirth-Schnatter and Regina Tüchler. "Bayesian parsimonious covariance estimation for hierarchical linear mixed models". In: *Stat. Comput.* 18.1 (2008), pp. 1–13. URL: https://link.springer.com/article/10.1007/s11222-007-9030-2.

[5] Sylvia Frühwirth-Schnatter and Helga Wagner. "Stochastic model specification search for Gaussian and partial non-Gaussian state space models". In: *J Econom.* 154.1 (2010), pp. 85–100. URL: https://doi.org/10.1016/j.jeconom.2009.07.003.

[6] A Gelman et al. *Bayesian Data Analysis*. 1995, pp. 134–141.

[7] Andrew Gelman, Donald B Rubin, et al. "Inference from iterative simulation using multiple sequences". In: *Stat Sci.* 7.4 (1992), pp. 457–472. URL: https://projecteuclid.org/euclid.ss/1177011136.

[8] Andrew Gelman, Daniel Simpson, and Michael Betancourt. "The prior can often only be understood in the context of the likelihood". In: *Entropy* 19.10 (2017), p. 555. URL: https://doi.org/10.3390/e19100555.

[9] Andrew Gelman et al. "Prior distributions for variance parameters in hierarchical models (comment on article by Browne and Draper)". In: *Bayesian Anal.* 1.3 (2006), pp. 515–534. URL: https://projecteuclid.org/download/pdf_1/euclid.ba/1340371048.
